# Transient human auditory cortex activation during volitional attention shifting

**DOI:** 10.1371/journal.pone.0172907

**Published:** 2017-03-08

**Authors:** Christian Harm Uhlig, Alexander Gutschalk

**Affiliations:** Department of Neurology, Ruprecht-Karls-Universität Heidelberg, Heidelberg, Germany; Harvard Medical School, UNITED STATES

## Abstract

While strong activation of auditory cortex is generally found for exogenous orienting of attention, endogenous, intra-modal shifting of auditory attention has not yet been demonstrated to evoke transient activation of the auditory cortex. Here, we used fMRI to test if endogenous shifting of attention is also associated with transient activation of the auditory cortex. In contrast to previous studies, attention shifts were completely self-initiated and not cued by transient auditory or visual stimuli. Stimuli were two dichotic, continuous streams of tones, whose perceptual grouping was not ambiguous. Participants were instructed to continuously focus on one of the streams and switch between the two after a while, indicating the time and direction of each attentional shift by pressing one of two response buttons. The BOLD response around the time of the button presses revealed robust activation of the auditory cortex, along with activation of a distributed task network. To test if the transient auditory cortex activation was specifically related to auditory orienting, a self-paced motor task was added, where participants were instructed to ignore the auditory stimulation while they pressed the response buttons in alternation and at a similar pace. Results showed that attentional orienting produced stronger activity in auditory cortex, but auditory cortex activation was also observed for button presses without focused attention to the auditory stimulus. The response related to attention shifting was stronger contralateral to the side where attention was shifted to. Contralateral-dominant activation was also observed in dorsal parietal cortex areas, confirming previous observations for auditory attention shifting in studies that used auditory cues.

## Introduction

In order to explore the neural basis of auditory perception, stimuli have been used whose perception is not fully determined by the physical stimulus. For example, the auditory stream segregation phenomenon can be used to create sound sequences that flip back and forth between the perception of one or two auditory streams [1,2]. When listeners indicate the time of their perceptual reversals by pressing a response button, an associated BOLD transient can be demonstrated in widespread parts of the auditory cortex [3,4]. This BOLD transient could be related to the perception of an auditory event, the timing of which is not determined by the physical stimulus, but a number of alternative interpretations are difficult to rule out. For example, it could be that the auditory cortex activation is related to a shift of attention preceding or following the change of perceptual organization.

It is well established that BOLD activity in the auditory cortex is enhanced [5,6], or modulated [7,8] by selective attention. In situations where one of two streams is selectively attended, electroencephalography (EEG) [9] and magnetoencephalography (MEG) [10,11] have been used to demonstrate that the phase-locked response to each single tone of the attended stream is enhanced. Without this temporal resolution, and without high spatial resolution to separate enhancement along frequency regions [12], the overall activation differences between distinct attention foci remain small in fMRI [13]. The overall enhancement of fMRI activation by attention towards sound is typically stronger with a contrast of auditory compared to visual attention [6,13], or auditory attention compared to rest [14,15].

For the understanding of bistable reversals, where attention is continuously maintained towards the auditory stimulation, the intra-modal reorienting of attention is more important. Previous studies have shown transient BOLD activity in the auditory cortex for cued, volitional shifting of attention from vision to audition [16], but not for shifts of attentional focus within the auditory modality [17]. This was subsequently confirmed by two studies of intra-modal auditory attention shifts [18,19]; these studies both found transient activity in the temporo-parietal junction, close to the planum temporale, but not in auditory cortex in and around Heschl's gyrus. Only when a similar task was used in EEG, an enhanced negative difference wave for intra-modal auditory orienting was observed, which may indicate enhanced auditory cortex activity [20], but the sources of the wave were not analyzed in more detail.

The nature of transient BOLD activity in sensory cortex associated with attention shifts [16] remains unknown at this point, and there are a number of possible functional associations: (1) It could be that the BOLD transient is related to enhanced sensory processing [5–11], which is stronger after the shift than in a continuous mode. (2) The BOLD transient could be related to the modulation of sensory processing, e.g. by top-down modulation, but independent of the sensory input. The transient nature might then be related to the switching or initiation of this attentional set [21] within the auditory cortex, or again the activity might simply be stronger at its beginning. (3) The BOLD transient might be overall unspecific and simply represent a general alerting/arousal within the auditory system.

Here, we reevaluate if BOLD transients are also associated with attention switching within the auditory modality. The problem of coupling the shift of attention with a sensory cue [16–19] is that it may independently influence activity in the auditory cortex: When the participant was cued by a visual stimulus to shift attention within the auditory modality, then enhanced activity in the auditory cortex may already be expected based on the shift of attention from the visual cue stimulus back to the auditory modality [15,16]. Conversely, auditory cue stimuli are readily expected to evoke transient BOLD activity in the auditory cortex related to sensory processing and likely also related to exogenous attention orienting, in particular when the cue sounds occur rarely [22]. To the degree that processing an auditory cue involves attentional resources that overlap with those required for shifting auditory attention, these setups could therefore miss attention-related activity in the auditory cortex that is necessarily required to shift auditory attention. This is because resources that are required for volitional attention shifting may not be exclusive for volitional attention shifting, but could instead be similarly recruited in other task contexts.

**Fig 1:**
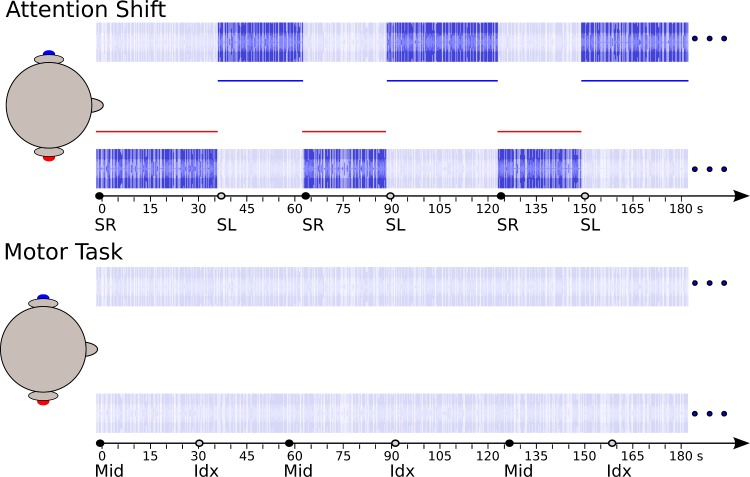
Schematic of the experimental paradigm. During both parts of the experiment, participants listened to an ongoing dichotic stimulation that was asynchronous between the ears. In the attention-shift experiment (upper panel), they were instructed to focus on the left- or right-ear stimulus in alternation, and indicate each shift of the attended side by a button press (SR, shift right; SL, shift left). In the control experiment (motor task, lower panel), participants were instructed to ignore the auditory stimulation and press the two response buttons in alternation at the same pace (Mid, middle finger; Idx, index finger).

To avoid these limitations in the present study, we explored if BOLD transients in the auditory cortex are induced by spontaneously shifting the focus of auditory attention, in the absence of any sensory cues or other task-relevant stimulus transients. We presented a continuous, dichotic stimulus that was configured such that two streams were generally perceived and attention could easily be shifted from one to the other stream without changing the perceptual organization. Listeners were instructed to continuously attend to one of the two streams. They were further instructed to shift their attention from the attended to the unattended stream after a while, then continue to attend this stream, and so on. These shifts in attention were self paced, and indicated to the experimenter by pressing one of two response buttons for the analysis of the associated BOLD activity (Fig 1). An attention shift from one to the other side was used, because we expected to find contralateral-dominant activity with this setup [19], in particular in the dorsal parietal lobe, which could be used as indirect confirmation of task compliance. A second experiment was performed to control for the role of the motor task and interval planning. Here, a control condition was added where the same stimuli were played, but participants were instructed to ignore the sound and press one of the two response buttons in alternation and at the same pace.

## Materials and methods

### Participants

42 healthy listeners participated in this study, 21 in each experiment. One listener from the first experiment and two from the second experiment were excluded from further analysis because of head movement in the scanner. The data of the remaining 20 listeners of experiment 1 (17 female, 3 male) with a mean age of 24.0 ± 3.3 years (standard deviation: S.D.) and 19 listeners in experiment 2 (14 female, 5 male) with a mean age of 24.3 years ± 3.1 years (S.D.) were fully analyzed. All listeners were compensated by an hourly payment; they had normal pure-tone audiograms with thresholds less than 10 dB HL between 0.125 and 12.5 kHz (one frequency at 15 dB HL was allowed) and provided written informed consent prior to their participation in the experiments. The experimental protocol was approved by the ethics committee of Heidelberg University Medical School.

### Stimuli

Stimuli were generated with MATLAB (The Mathworks, Natick, MA, USA) and stored as wave files with a sample-rate of 48 kHz. The wave files were presented via a D/A converter and headphone amplifier (MR Confon; MR confon GmbH, Magdeburg, Germany) with Sensimetrics S14 in-ear headphones (Sensimetrics Corporation, Malden, MA, USA). The non-linear transfer function of the in-ear headphones were corrected using the wave-file converter software provided by the manufacturer.

The stimuli consisted of sequences of amplitude-modulated (AM) pure tones with a duration of 60 ms, including 20-ms cos-shaped ramps. 2.2 kHz pure tones, with a 21 Hz AM were presented to the left, and 4.4 kHz pure tones with a 42 Hz AM were presented to the right. The modulation depth was 0.8. The inter-stimulus interval (ISI) was randomized between 10 ms and 60 ms, independently for the left- and right-ear stream to avoid any rhythmical relationship between the two streams. In the first experiment, four runs of 9:20 min duration were presented. Six runs of the same stimuli were presented in experiment 2. Additionally, an auditory-cortex localizer with 5.7 s long sounds of the same configuration followed by 16 s silent periods was presented in experiment 1. After experiment 1 had revealed consistent activation of auditory cortex at the vertex level, similar results for the functional localizer and the anatomical ROIs, the localizer was not presented in experiment 2 to reduce the session duration.

### Procedures

For both experiments, the tasks were explained one day before the fMRI session, including one or two training runs with circumaural headphones connected to a desktop computer.

In-house software was used for stimulus presentation and collection of listeners' responses with a LUMItouch optical response keypad (Photon Control, Burnaby, BC, Canada).

## Experiment 1: Attention shifting

The task for the first experiment was to keep listening to either the left or the right sound stream and shift the focus of attention approximately two times per minute to the opposite stimulus, listen to this stimulus, and so on. Participants were instructed not to count seconds or perform other tasks beside keeping attention to the chosen sound source. They indicated the attention shift by pressing a button, with their right index finger (Idx) for a shift to the left side and with their right middle finger (Mid) for a shift to the right side in the moment of shifting. During the whole time, listeners were instructed to keep their eyes still and fixate a line at the top of the scanner bore, straight ahead.

## Experiment 2: Attention shifting and motor control task

In experiment 2, a self-paced motor task was added as control, where listeners simply pressed the two response buttons in alternation. They were instructed to press the buttons about two times per minute without paying attention to the auditory stimulation and without performing any other tasks, like counting etc. Like in the attention task, they were instructed to fixate the line in front of them. In the other half of the runs, the task instruction of experiment 2 was identical to the attention shifting task of experiment 1. Six runs were performed. Attention shifting was performed in runs 1, 3, and 5. The self-paced motor task was performed in runs 2, 4, and 6.

### Imaging

All MRI data were acquired with a 3 Tesla Tim-Trio scanner (Siemens, Erlangen, Germany), equipped with a 32-channel, phased-array head coil. Two (to achieve a better SNR for the surface reconstruction) T1-weighted magnetization-prepaired rapid gradient echo sequences (MPRAGE) were acquired with a dimension of 256 x 256 x 192 voxel, an isovoxel resolution of 1 mm^3^, a TR of 1570 ms, a TE of 2.63 ms, a TI of 900 ms and a Flip Angle of 9 degrees. These scans were used to place the functional volume, which included the whole brain. The functional volume for both experiments comprised 32 slices (4 mm thickness, distance 33%) with a field of view of 192 x 192 mm (64 x 64 voxel, resolution of 3 x 3 mm). The parameters for BOLD imaging were repetition time (TR) = 2 s, echo time (TE) = 30 ms, and a Flip Angle of 80 degrees. For the acquisition of the auditory-cortex localizer in experiment 1, a TR of 8 s was used while the acquisition was clustered to 1.6 s to provide 6.4 s long intervals without gradient noises [23,24]. All other parameters were as described above.

### Data analysis

#### Activation maps

The structural data were analyzed with FreeSurfer (http://surfer.nmr.mgh.harvard.edu/) Version 5.3.0 using the surface-based stream [25,26]. The functional data were motion corrected and aligned to a template brain using FreeSurfer on an Ubuntu 12.04 LTS 64 bit operating system with an Intel Core i5-2500 CPU @ 3.3GHz x 4 processor. The paradigm file for each run of each listener was based on the trigger times saved by the presentation and feedback software and the scanning times stored in the DICOM files. The duration of the events–attention shifts and button presses–was arbitrarily set to 1 s as approximated duration used for the cross-correlation analysis. Considering that the mental event starts before the button press, event times were set to 600 ms before the registered button presses, the approximate delay observed between the onset of the prominent BP2 component of the Bereitschaftspotential and the subsequent motor task [27]. The individual average signal-intensity maps in the main experiments were calculated for the Attention-Shift-versus-Baseline contrast, and for the Motor-Task-versus-Baseline. The baseline was defined as the total interval in between the respective events. The additional auditory localizer stimulus in experiment 1 was evaluated by calculating the Sound-versus-Silence contrast; the localizer was based on the second level (group) analysis in a template brain surface and was then projected back to the signal space of the individual participants. Therefore, the same localizer could also be used for a different group of participants in experiment 2. Slice-timing correction was used to account for different acquisition times within the functional volume. The individual contrasts were used to perform a mixed effects group analysis, corrected for multiple comparisons with the false-discovery-rate [28] method and a corrected cutoff of p < 0.05.

#### Region of interest based analysis

Region of interest (ROI) based analyses were performed to (1) evaluate response lateralization within the auditory cortex and (2) to compare the attention-shift and motor-task contrasts. ROIs were defined as surface labels in FreeSurfer, which were used to extract the beta values from the first level analysis with combined FreeSurfer and MATLAB scripts. Two different ROI definitions were used for the auditory cortex: One was based on the group-level analysis of the functional localizer from experiment 1 (AudF) using a vertex-wise threshold of p < 0.00001 and a cluster-wise correction for multiple comparisons with p < 0.0001. This conservative threshold was chosen to limit the ROI to the region most robustly responsive across participants. The other two were anatomically defined. Heschl's gyrus (HG) up to (but not including) the fusion with the superior temporal gyrus (STG) was used as estimate of the auditory core area. The Planum temporale (PT) was separately analyzed as area that comprises predominantly auditory belt cortex, and was defined as region within the borders of HG, STG, and the inferior parietal gyrus.

Each ROI was separately evaluated with an analysis of variance (ANOVA) for repeated measures using R Software [29]. To evaluate response lateralization in dependence of the task, the factors hemisphere and direction of attention shift (shift to left: SL, shift to right: SR) were used.

To compare attention shifts and motor task in experiment 2, behavioral condition (attention shifts versus motor task) was added as third factor to the factors hemisphere and direction of attention shift, respective finger used (SL versus SR / index finger (Idx) versus middle finger (Mid)). Both analyses used the baseline-referenced beta values of each condition. The a priori hypothesis of enhanced, contralateral dominant activity in the auditory cortex was separately tested in all three ROIs with a significance level adjusted to p < 0.01.

To evaluate the second a priori hypothesis, i.e. that a transient response evoked by the pure motor action was significantly different from baseline in auditory cortex, a two way ANOVA with the factors pure motor action (button press versus baseline) and hemisphere was additionally performed. This analysis is not reported in detail, because auditory cortex activation was already significant in the voxel-wise analysis for this contrast.

Additionally to the a priori hypotheses in the auditory cortex, response lateralization and enhancement in the attention compared to the control task were evaluated in 10 additional ROIs (taken from the FreeSurfer Destrieux Atlas [30]) to control for expected activation patterns and to compare our results with previous studies. Activity in the primary visual (calcarine sulcus [S_calcarine]) and somatosensory cortex (postcentral gyrus [G_postcentral]) were included to evaluate if the observed effects were exclusive to the auditory cortex or also observed in other sensory regions. Motor cortex (precentral gyrus [G_precentral]) was included to probe for the expected activation contralateral to the response hand. A number of dorsal parietal areas (precuneus [G_precuneus], superior parietal gyrus [G_parietal_sup], and intraparietal sulcus [S_intrapariet_and_P_trans]), the temporo-parietal junction (supramarginal gyrus [G_pariet_inf-Supramar]), pre-frontal areas (superior [S_precentral_sup_part] and inferior [S_precentral_inf_part] precentral sulcus), and the insula ([S_circular_insula_sup]) were included because of their expected role for attention shifting observed in previous studies [16–19]. The significance level for the ROI analysis outside of auditory cortex was set to p < 0.005.

#### Reconstruction of the BOLD-signal response function

To explore the time course of the BOLD response for attention shifts in auditory cortex, the response function was reconstructed for the AudF ROI. To this end, the time series were linearly interpolated to a uniform sample rate of 1 Hz relative to the time of the button presses. The interpolated data were then averaged across trials, separately for each of the four conditions (left/right attention shift, left/right button press).

## Results

### Behavioural data

In experiment 1, the mean duration for which listeners maintained their attention focused to the right, between two attention shifts, was 38,6 s ± 3,1 s (S.D.) and the duration that they maintained their attention focused to the left was on average 39,2 s ± 3,7 s (S.D.). The average number of shifts was 66,7 ± 1,0 (S.D.).

In the experiment 2, the mean interval duration for attention to the left was 36,8 s ± 2,5 s (S.D.) and for attention to the right 36,9 s ± 2,4 s (S.D.). The average time interval between button presses was 31,0 s ± 2,9 s (S.D.) following a response with the index finger, and 31,8 s ± 2,6 s (S.D.) following a response with the middle finger. The average number of shifts was 48,9 ± 0,4 (S.D.) and the average number of motor activations was 62,5 ± 1,0 (S.D.).

### Activation maps

**Fig 2:**
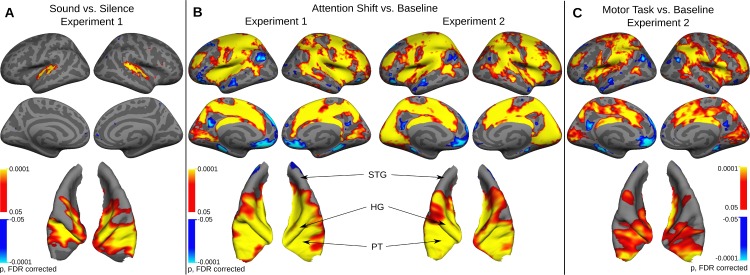
Group activation maps evoked by attention shifts and button presses (N = 20 in experiment 1; N = 19 in experiment 2) projected on whole brain views of the FreeSurfer template brain and a detailed view of auditory cortex region. **(A)** The Sound versus Silence contrast of the auditory localizer reveals activation confined to auditory cortex. **(B)** The Attention-Shift-versus- Baseline contrast reveals wide-spread activity including auditory cortex. **(C)** The Motor Task-versus-Baseline contrast reveals a similar pattern of activation, which appears to be somewhat weaker in some areas. Abbreviations: STG: Superior temporal Gyrus; HG: Heschl's Gyrus; PT: Planum temporale.

Fig 2A shows activity in the auditory cortex, as estimated by the passive sound-versus-silence contrast of the functional localizer. As can be seen in Fig 2B, these areas were also active when a shift of attention was indicated by a button press. Note that this contrast is versus a baseline of ongoing stimulation and maintained, focused attention, and can therefore not be attributed to the acoustic stimulation or auditory attention per se. As expected, activation is not limited to the auditory cortex for this contrast, but shows activation of a wide-spread cortical network that also includes the posterior superior temporal sulcus, major parts of the parietal cortex (inferior parietal cortex, superior parietal cortex, intraparietal sulcus, and precuneus), frontal cortex (precentral gyrus and sulcus, supplementary motor area, dorsolateral frontal cortex), the anterior insular cortex, the cingulate gyrus, and occipital cortex (cuneus and calcarine sulcus).

The self-paced control task in Fig 2C shows overall less prominent activation, but in a similar extensive network. Most prominent is the activation in the inferior parietal lobe up to the intraparietal sulcus, in the anterior insular cortex, and in the dorso-lateral prefrontal cortex. There is also clear activity in the auditory cortex, including Heschl's gyrus and the planum temporale.

### Region-of-interest analysis (auditory cortex)

The planned ROI analysis in auditory cortex was performed to evaluate two aspects of the data: First, we tested the hypothesis that activity was more strongly enhanced contralateral to the attentional shift. Second, we tested if activity in auditory cortex was stronger for attention shifts compared to pure self-initiated button presses. Note that the latter comparison is not referenced against the same baseline; while the attention-shift condition is referenced against continuously maintained auditory attention, the motor control task is referenced against fixation.

**Fig 3:**
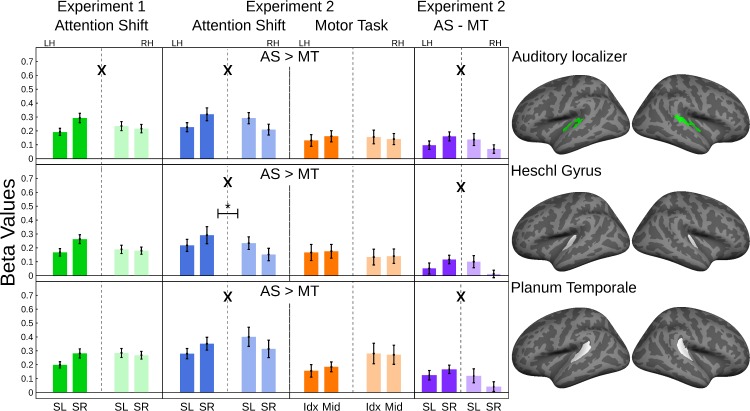
ROI Analysis of Sensory Cortex areas (N = 20 in experiment 1; N = 19 in experiment 2). Bar graphs: The ordinates show the regression-coefficients with standard errors for each ROI. The abscissa indictates the direction of the attention shifts (SL: Shift to the left; SR: Shift to the right), respective the used finger for the button presses (Idx: Index Finger; Mid: Middle Finger), and the hemisphere (LH: left hemisphere, RH: right hemisphere). Each line belongs to one ROI, which is shown in the right-most column. Main effects of hemisphere or condition are indicated with '*', interactions of hemisphere x condition are indicated with 'X'. Differences between activation for attention shifts (AS) and motor task (MT) are indicated by '>'. The significance levels are indicated as p<0.01 (*, X, >).

The numerical values shown in Fig 3 demonstrate that average activity within each auditory cortex is higher for an attention shift away from the hemisphere, and that activity is generally higher for attention shifts compared to the motor control task. The statistical analysis confirms stronger activity for attention shifts in experiment 2, and the significant interaction of hemisphere x shift x condition confirms the lateralization effect for the difference of attention shifts minus motor control (Table 1, Fig 3) in all auditory cortex ROIs (p < 0.01). The direction-dependent hemispheric lateralization within each condition (interaction of hemisphere x shift direction) was also significant for all three auditory cortex ROIs in experiment 2 (Fig 3) and for the functional ROI in experiment 1. As a limitation, it appears that in experiment 1 the latter effect was mostly driven by the left auditory cortex.

**Table 1:** ANOVAs for the auditory ROIs shown in Fig 3.

ROI	Exp.	Behavioural condition		Behavioural condition x direction of shift x hemisphere		Direction of shift x hemisphere		Hemisphere	
		p	(dF), F	p	(dF), F	p	(dF), F	p	(dF), F
**Auditory Localizer**	°AS					0.00586 *	(1,18), 9.627	0.459	(1,18), 0.571
	°°AS	0.0000329 *	(1,19), 30.08	0.00805 *	(1,19), 8.873	0.0000905 *	(1,19), 25.12	0.306	(1,19), 1.11
	°°MT					0.012	(1,19), 7.799	0.906	(1,19), 0.014
**Heschl Gyrus**	°AS					0.0288	(1,18), 5.599	0.109	(1,18), 2.829
	°°AS	0.00724 *	(1,19), 9.125	0.000812 *	(1,19), 16.12	0.000364 *	(1,19), 19.16	0.00335 *	(1,19), 11.41
	°°MT					0.889	(1,19), 0.02	0.0452	(1,19), 4.633
**Planum Temporale**	°AS					0.0246	(1,18), 5.959	0.154	(1,18), 2.204
	°°AS	0.000096 *	(1,19), 24.85	0.00507 *	(1,19), 10.18	0.000102 *	(1,19), 24.58	0.297	(1,19), 1.153
	°°MT					0.0629	(1,19), 3.931	0.0216	(1,19), 6.325

N = 19 subjects in experiment 1 (°AS) and N = 20 subjects in experiment 2 (°°AS: attention shift, °°MT: motor task). The factors hemisphere and direction of shift were used in experiment 1 (two-way ANOVA). In experiment 2 the factors hemisphere, direction of shift, and behavioural condition were entered (three-way ANOVA). The interaction of all three factors is equivalent to the direction of shift x hemisphere interaction for the difference of AS-MT (cf. Fig 3, column 4). Significant tests (p<0.01) are highlighted with a star (*).

**Fig 4:**
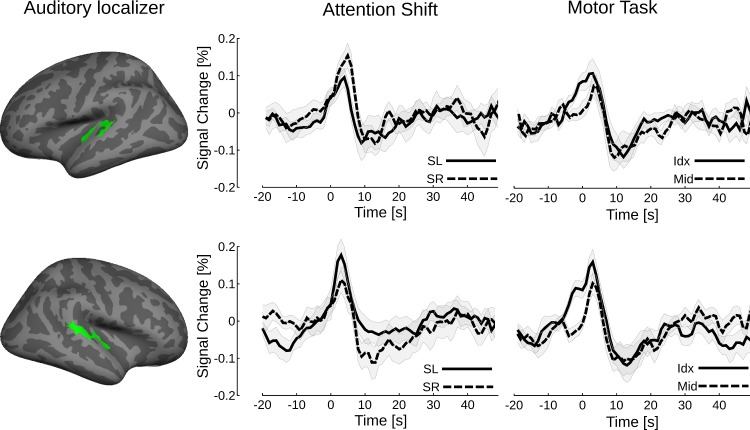
BOLD time courses in the auditory cortex for experiment 2 (mean ± standard error, N = 19). The time axis is relative to the buttonpress. The peak latency of the BOLD transient was for the attention shift to the left in right AC: 4.0 s ± 2.0 s, in left AC: 3.3 s ± 2.6 s; for the shift to the right in right AC: 3.8 s ± 2.0 s, in left AC: 3.6 s ± 2.3 s. For the motor task the peak latency was for the middle finger in the right AC 3.6 s ± 1.6 s, in the left AC: 4.1 s ± 2.2 s and for the index finger in the right AC: 3.1 s ± 1.8 s, in the left AC: 2.9 s ± 2.6 s.

The transient nature of the activation for attention shifts and button presses is confirmed by the reconstruction of the BOLD signal time course shown in Fig 4 for the functional auditory cortex ROI. The latency of the response was in the range of 3 to 4 s after the registration of the button press. Moreover, we evaluated if the contralateral dominance of the transient activity during attention shifts was related to potential lateralization of sustained BOLD activity in the time intervals before and after the shifts, where the listeners were instructed to pay sustained attention and which was used as baseline for the main analysis. The analysis revealed no lateralization in a 6-s interval before the shift. In an interval 6 s before and after the shift, a trend for attention-dependent lateralization was only observed in the right auditory cortex, however, this sustained lateralization was a factor of five (or more) smaller than the lateralization during attention shifts. No such attention-dependent lateralization was observed in the left auditory cortex. It can therefore be excluded that the attention-dependent lateralization during attention shifting simply reflects the transition between two opposite sustained patterns of lateralized activity. Similar observations were made in the other ROIs.

### Sensory and motor cortex

**Fig 5:**
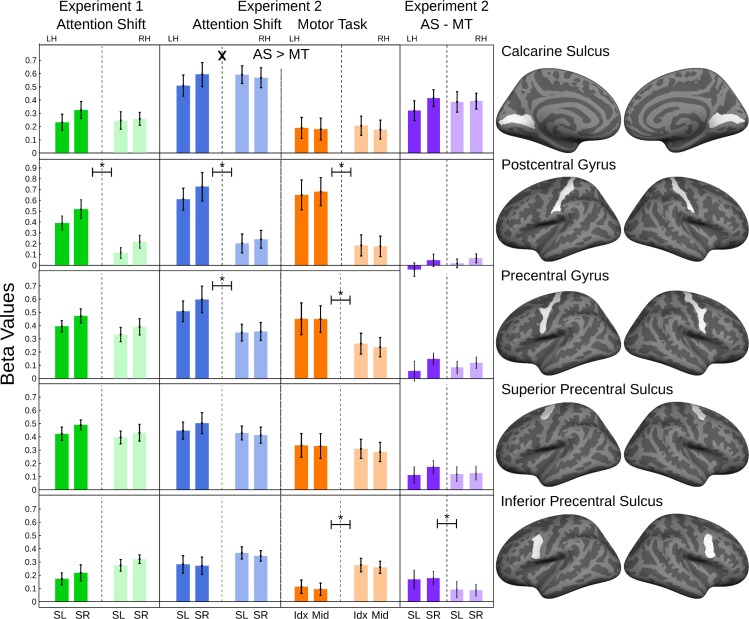
ROI analysis of frontal cortex areas and calcarine sulcus (N = 20 in experiment 1; N = 19 in experiment 2). Bar graphs: The ordinates show the regression-coefficients with standard errors for each ROI. The abscissa indictates the direction of the attention shifts (SL: Shift to the left; SR: Shift to the right), respective the used finger for the button presses (Idx: Index Finger; Mid: Middle Finger), and the hemisphere (LH: left hemisphere, RH: right hemisphere). Each line belongs to one ROI, which is shown in the right-most column. Main effects of hemisphere or condition are indicated with '*', interactions of hemisphere * condition are indicated with 'X'. Differences between activation for attention shifts (AS) and motor task (MT) are indicated by '>'. The significance levels are indicated as p<0.005 (*, X, >).

To explore if the lateralization observed for auditory attention shifting was also observed in other sensory areas, we performed a similar analysis for primary visual and somatosensory cortex. Activity in primary visual cortex, as evaluated with the calcarine-sulcus ROI (Fig 5, Table 2), was stronger in the attention shift compared to the control task. There was a significant interaction of hemisphere x shift direction for the attention shift task, but the hemisphere x shift direction x condition interaction only showed a non-significant trend.

**Table 2:** ANOVAs for the non-auditory ROIs shown in Fig 5.

ROI	Exp.	Behavioural condition		Behavioural condition x direction of shift x hemisphere		Direction of shift x hemisphere		Hemisphere	
		p	(dF), F	p	(dF), F	p	(dF), F	p	(dF), F
**Calcarine Sulcus**	°AS					0.0144	(1,18), 7.248	0.297	(1,18), 1.15
	°°AS	0.0000038 *	(1,19), 42.77	0.0167	(1,19), 6.954	0.000298 *	(1,19), 19.95	0.682	(1,19), 0.174
	°°MT					0.285	(1,19), 1.213	0.851	(1,19), 0.036
**Postcentral Gyrus**	°AS					0.658	(1,18), 0.203	0.00373 *	(1,18), 10.92
	°°AS	0.526	(1,19), 0.417	0.524	(1,19), 0.423	0.184	(1,19), 1.911	0.000000132 *	(1,19), 69.78
	°°MT					0.296	(1,19), 1.159	0.000000278 *	(1,19), 62.89
**Precentral Gyrus**	°AS					0.708	(1,18), 0.144	0.233	(1,18), 1.519
	°°AS	0.0162	(1,19), 7.036	0.228	(1,19), 1.555	0.0608	(1,19), 4.002	0.000286 *	(1,19), 20.12
	°°MT					0.173	(1,19), 2.013	0.000931 *	(1,19), 15.63
**Superior Precentral Sulcus**	°AS					0.308	(1,18), 1.095	0.352	(1,18), 0.909
	°°AS	0.0091	(1,19), 8.539	0.0553	(1,19), 4.2	0.0107	(1,19), 8.095	0.202	(1,19), 1.757
	°°MT					0.393	(1,19), 0.766	0.397	(1,19), 0.752
**Inferior Precentral Sulcus**	°AS					0.989	(1,18), 0	0.0358	(1,18), 5.105
	°°AS	0.00591	(1,19), 12.5	0.702	(1,19), 0.151	0.623	(1,19), 0.251	0.0804	(1,19), 3.433
	°°MT					0.927	(1,19), 0.009	0.000511 *	(1,19), 17.84

N = 19 subjects in experiment 1 (°AS) and N = 20 subjects in experiment 2 (°°AS: attention shift, °°MT: motor task). The factors hemisphere and direction of shift were used in experiment 1 (two-way ANOVA). In experiment 2 the factors hemisphere, direction of shift, and behavioural condition were entered (three-way ANOVA). The interaction of all three factors is equivalent to the direction of shift x hemisphere interaction for the difference of AS-MT (cf. Fig 5, column 4). Significant tests (p<0.005) are highlighted with a star (*).

Activity in the post-central gyrus, as estimate for the primary somatosensory cortex, reflects the motor task and the associated afferent control rather than attention: First, the responses are stronger on the left, contralateral to the response hand. Second, there was no significant difference between attention shifts and the button-press control. Third, a similar pattern was observed in motor cortex in the pre-central gyrus ROI.

### Frontal, insular, and parietal cortex

Next, we evaluated if the network that has been previously shown to be involved in directing attention [17,31] was differentially activated for the attention-shift compared to the control task. Moreover, we tested if the activation in these areas showed contralateral dominance [19], as reported above for the auditory cortex.

All evaluated ROIs showed prominent activation across conditions, as was readily expected based on the activation maps shown in Fig 2. While numerical and statistical trends for a stronger response in the attention shift compared to the control task in experiment 2 were present in all frontal and parietal ROIs, the conservative threshold chosen because of the multiple ROIs in this exploratory analysis (p < 0.005) was only reached in the precuneus.

**Fig 6:**
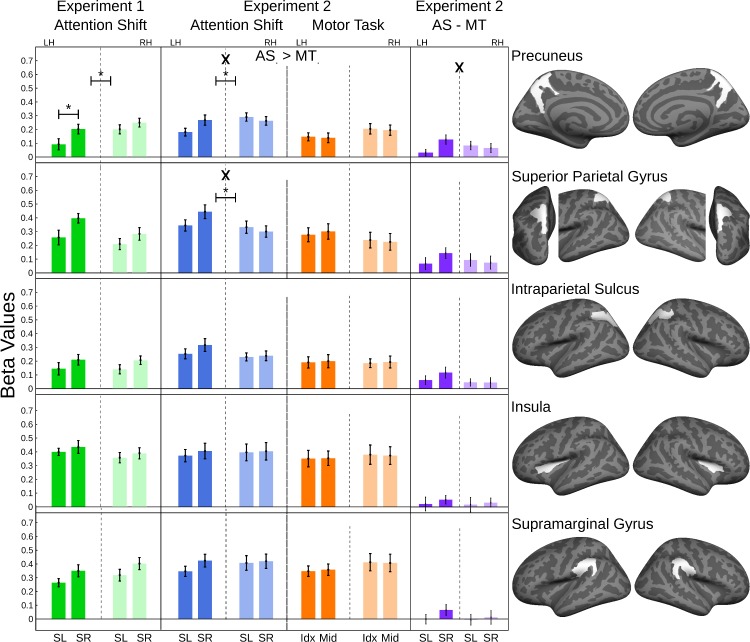
ROI analysis of association cortex areas. (N = 20 in experiment 1; N = 19 in experiment 2). Bar graphs: The ordinates show the regression-coefficients with standard errors for each ROI. The abscissa indictates the direction of the attention shifts (SL: Shift to the left; SR: Shift to the right), respective the used finger for the button presses (Idx: Index Finger; Mid: Middle Finger), and the hemisphere (LH: left hemisphere, RH: right hemisphere). Each line belongs to one ROI, which is shown in the right-most column. Main effects of hemisphere or condition are indicated with '*', interactions of hemisphere * condition are indicated with 'X'. Differences between activation for attention shifts (AS) and motor task (MT) are indicated by '>'. The significance levels are indicated as p<0.005 (*, X, >).

Similarly, contralateral dominance, as indicated by an interaction of shift direction x hemisphere x condition was only significant in the precuneus (Fig 6, Table 3). (Trends for a similar pattern were observed in the superior parietal gyrus, the intraparietal sulcus, and the superior precentral sulcus).

**Table 3:** ANOVAs for the non-auditory ROIs shown in Fig 6.

ROI	Exp.	Behavioural condition		Behavioural condition x direction of shift x hemisphere		Direction of shift x hemisphere		Hemisphere	
		p	(dF), F	p	(dF), F	p	(dF), F	p	(dF), F
**Precuneus**	°AS					0.00735	(1,18), 9.004	0.00351 *	(1,18), 11.1
	°°AS	0.00167 *	(1,19), 13.63	0.0000226 *	(1,19), 32.07	0.0000318 *	(1,19), 30.26	0.00355 *	(1,19), 11.24
	°°MT					0.851	(1,19), 0.036	0.0136	(1,19), 7.476
**Superior Parietal Gyrus**	°AS					0.0194	(1,18), 6.525	0.0209	(1,18), 6.348
	°°AS	0.017	(1,19), 6.916	0.00575	(1,19), 9.814	0.00167 *	(1,19), 13.63	0.00107 *	(1,19), 15.16
	°°MT					0.0321	(1,19), 5.3963	0.0141	(1,19), 7.394
**Intra-Parietal Sulcus**	°AS					0.999	(1,18), 0	0.918	(1,18), 0.011
	°°AS	0.00637	(1,19), 9.526	0.0494	(1,19), 4.439	0.051	(1,19), 4.371	0.11	(1,19), 2.834
	°°MT					0.99	(1,19), 0	0.841	(1,19), 0.041
**Insula**	°AS					0.925	(1,18), 0.009	0.226	(1,18), 1.565
	°°AS	0.343	(1,19), 0.948	0.503	(1,19), 0.467	0.118	(1,19), 2.702	0.634	(1,19), 0.235
	°°MT					0.568	(1,19), 0.338	0.345	(1,19), 0.939
**Supra-Marginal Gyrus**	°AS					0.934	(1,18), 0.007	0.0422	(1,18), 4.744
	°°AS	0.544	(1,19), 0.382	0.155	(1,19), 2.207	0.0908	(1,19), 3.194	0.331	(1,19), 0.997
	°°MT					0.284	(1,19), 1.221	0.18	(1,19), 1.945

N = 19 subjects in experiment 1 (°AS) and N = 20 subjects in experiment 2 (°°AS: attention shift, °°MT: motor task). The factors hemisphere and direction of shift were used in experiment 1 (two-way ANOVA). In experiment 2 the factors hemisphere, direction of shift, and behavioural condition were entered (three-way ANOVA). The interaction of all three factors is equivalent to the direction of shift x hemisphere interaction for the difference of AS-MT (cf. Fig 6, column 4). Significant tests (p<0.005) are highlighted with a star (*).

Two other patterns observed are mentioned for completeness: A significant hemisphere effect was observed in the inferior pre-central sulcus: here, activity was significantly stronger on the right during the motor task. This leads to a significant hemisphere x condition interaction that is caused by left-hemisphere activity being stronger in the attention shift compared to the control condition. A similar pattern was not observed in any of the other ROIs. Finally, the insula and marginal gyrus ROI where distinct in so far, as they did not show any statistical or relevant numerical trend for a difference between attention and control conditions, despite high overall activity in these regions.

## Discussion

The results of this study suggest that self-initiated attention shifts within the auditory modality are associated with transient BOLD activity in auditory cortex, including most of Heschl's gyrus and planum temporale. This activation appears to comprise two components; one that is specifically related to the target of auditory attention, and another one that is less specific and potentially related to alerting/arousal. We will first discuss these two components in the auditory cortex, and will then briefly comment on other brain areas.

The transient BOLD response observed time locked to volitional auditory attention shifts in auditory cortex confirms our hypothesis that auditory attentional reorienting evokes transient enhancement not only in supra-modal [16,18,19], but also in modality specific cortex. The reason for the lack of a similar finding in previous studies of auditory attention shifting [17–19] is most likely related to the auditory cue that was generally coupled to the attention shift in these studies (cf. introduction). Salient, rare auditory cues are expected to evoke prominent auditory cortex activation even outside of the focus of attention [22], probably by drawing attentional resources via the ventral attention system [32]. If the same or at least similar resources are required for volitional attentional reorienting, this activation would be subtracted out by modelling or subtracting the activity evoked by the cue stimulus in a control context.

Because the attention shifts were not cued in the present experiment, a motor response was used instead to model the time at which the attention shift occurred. Based on the contrast between the attention shift and the motor control, a specific, contralateral-dominant component could be separated in the context of attention shifts to the opposite ear. It is well established that continuous attention to sound enhances BOLD activity in auditory cortex in comparison to e.g. a visual-attention condition [6]. However, the contrast between conditions where attention is focused to the left versus the right has produced mixed results in the past. Two studies reported stronger enhancement in the auditory cortex contralateral to the attentional focus [33,34]. One possibility is therefore that enhanced contralateral auditory cortex activity during attention shifts is related to the continuous enhancement following the attention shift, and for example decreases under continuous maintenance of attention, producing a transient BOLD characteristic at the time of the attention shift. Alternatively, it could be that the lateralized transient is more specifically related to one of the stages of attentional orienting, in particular to shifting attention or to engaging with the new stimulus, as suggested [35] for the visual system. At this point, the available data cannot dissociate between these alternative explanations.

The second component of transient auditory-cortex activation in the attention-shifting task was similarly observed for the self-initiated button presses, which were added in experiment 2 as a control task. More readily expected, the control task produced activation in the task-positive network [36–38]. One possible explanation for the non-specific transient in auditory cortex could thus be general alerting (arousal) that is triggered by the occasional decisions to perform a button press or an attention shift, or by the execution thereof. Neither the task-positive nor the default-mode network have so far been reported to involve activity of auditory (or more generally sensory) cortex. In mouse models, however, it has been shown that arousal–indexed by pupil dilation and ripple activity in the hippocampus–is closely correlated with membrane potentials in auditory cortex [39]. Moreover, phasic pupil dilations are not only preceded by activity in the locus coeruleus, but also by activity in the inferior colliculus [40], from where it could potentially be transferred to the auditory cortex independently or additionally to projections emanating from the locus coeruleus. In human fMRI, phasic pupil dilation has been shown to be associated with increased activity in the default-mode network, whereas BOLD activity in sensory areas was reduced [41]. In a study that explored the influence of alerting on auditory and visual detection, however, it was found that sensory areas showed enhanced, modality specific activity for trials with faster responses in addition to regions of the default-mode network [42]. We therefore think that alerting (arousal) is one potential mechanism that could trigger the non-specific component of the transient, task-related activity in auditory cortex found in the present study. Previous data did not show auditory cortex activation during button presses [19,42]. Possibly, the effect observed in the present study was partly context dependent and related to the interleaved auditory task context, such that listeners could not completely avoid auditory attention during button pressing. Alternatively, it may suffice that the control task itself was not demanding enough to distract attention away from audition.

There are a number of less likely explanations for the auditory cortex activity observed in the control task, which we briefly discuss here for completeness: While the sound of the response key was clearly masked in our setting, we cannot exclude that listeners occasionally generated sounds themselves that are temporally associated with the motor task, but such sounds would be expected to be rather inconsistent. The comparatively short latency of the BOLD response, about 3 to 4 s after the button press, rather suggests that the response is not in response to (self-generated) sound, but related to the task more directly.

A parallel to the observation in the present study comes from studies in awake monkeys, where unit responses in auditory cortex have been reported to respond time-locked to a visual cue and to the touch of a response bar, when the monkeys performed an auditory task that they had been trained on for a long time [43]. Similarly, in intracranial recordings from Heschl's gyrus in patients with epilepsy, activity around the response latency has been reported in the context of an auditory task [44]. A potential neural explanation of this phenomenon is that corollary discharges (efferent copies) are the source of auditory cortex activation. In an fMRI study that compared passive and self generated sounds, stronger BOLD activity was observed for self-generated sound, and more so in the AC contralateral to the hand used [45,46]. However, the circuit between motor cortex and AC has recently been shown to be inhibitory [47], and human studies using MEG showed smaller responses for self-generated compared to passively presented sounds [48], matching better to the BOLD deactivation in auditory cortex observed in the context of visual target detection [42].

Activity in primary visual cortex, in contrast to primary somatosensory cortex, also showed task dependent enhancement, similar to the effect observed in auditory cortex. Potential reasons could be a general coupling of auditory and visual attention orienting [49,50], or small eye movements (or saccade suppression) in the context of auditory attention reorienting [51,52]. In visual cortex, BOLD transients have previously been observed in the context of task responses without associated visual stimulation [53]. This effect might potentially be related to the visual cortex coactivation that was observed here. Conversely, it is unlikely that the unspecific transient in auditory cortex observed here is an auditory variant of the phenomenon in vision [53], given that activity in primary visual cortex has previously been observed in the context of auditory target detection, whereas detection of visual targets–including a button press–were associated with reduced activity in auditory cortex [42].

The analysis of extra-auditory areas confirmed key results of previous fMRI studies of auditory intramodal attention shifting [17–19]. While part of this activation is likely unspecific task-related activity [38], the contralateral dominance of activity in the dorsal parietal lobe reflects the direction of the self-initiated attention shifts [19]. This finding provides an indirect confirmation that our listeners performed the switching task correctly, which is important given the lack of a behavioral task control. It has recently been suggested that activity in the dorsal parietal lobe is related to eye movements in the direction of the auditory attention, or to saccade suppression, rather than to the orienting of auditory attention [52]. While we instructed our participants to fixate, we did not apply eye-tracking and can, therefore, not rule out that small eye movements were associated with reorienting of attention. Thus, while we cannot comment on the functional role of the activity in the dorsal parietal lobe for audiory attention, its lateralization still confirms task compliance. While we consider it unlikely that the activity in auditory cortex reported here is strongly related to eye movements, eye position has been shown to modulate activity in the auditory cortex [54], and a contribution of eye movements on the transient BOLD activity in auditory cortex cannot be excluded at this point.

Our results showed only a trend for stronger activity in the precentral frontal cortex for attention shifts, which would generally be in line with previous studies [14,16,17,19,22,22,55,56]. Attention-specific activity in the temporo-parietal junction, which had been observed by two studies of cued auditory attention shifting [19,55], was not confirmed here. While there was transient activity in the temporo-parietal junction (supramarginal gyrus ROI) in the present study as well, there was no hint of a difference between attention shifting and motor control tasks. This does certainly not rule out a role of this area for auditory attention shifting, but it could indicate that its role is less specific for self-initiated attention shifting than had been previously thought [19].

## Conclusion

The findings of this study suggest that auditory cortex is not only activated by exogenous, but also by endogenous orienting of attention. These findings have important implications for the study of transient auditory events that are, for example, linked to reversals of bistable auditory percepts [3,4]. While the transient activation in auditory cortex observed in these contexts might still be related directly to the perceptual event or to exogenous orienting triggered by the event, the activation pattern cannot be easily dissociated from less specific endogenous events preceding the reversal, or from more general alerting effects.
